# virMine 2.0: Identifying Viral Sequences in Microbial Communities

**DOI:** 10.1128/mra.00107-22

**Published:** 2022-05-02

**Authors:** Genevieve Johnson, Catherine Putonti

**Affiliations:** a Bioinformatics Program, Loyola University Chicago, Chicago, Illinois, USA; b Department of Biology, Loyola University Chicago, Chicago, Illinois, USA; Indiana University, Bloomington

## Abstract

Here, we present virMine 2.0, the next generation of the virMine software tool. virMine 2.0 uses an exclusion technique to remove nonviral data from sequencing reads and scores the remaining data based on relatedness to viral elements, eliminating the sole dependency on homology identification.

## ANNOUNCEMENT

In contrast to the wealth of data available for cellular organisms, the viral diversity on Earth is underrepresented in sequence databases. As a result, homology-based identification of viral sequences is limited. Furthermore, viruses contain a high degree of genetic diversity, and it can be nearly impossible to distinguish conserved genes among viruses (1). Bioinformatic approaches for *de novo* viral identification employ homology-based, nucleotide usage, or coverage analyses or combinations thereof (for a review, see reference 2).

Previously, we introduced a tool called virMine (3), which utilizes the wealth of sequence data for cellular organisms to identify likely viral sequences in metagenomes. The tool takes either (i) short reads, either single-end or paired-end fastq file(s), or (ii) a long-read or assembled-sequence fasta file. In the former case, read quality control is conducted, followed by assembly. Three methods for assembly are included in virMine, namely, SPAdes, metaSPAdes, and MEGAHIT; alternatively, the user can select the all3 option, in which all three assembly methods are executed and the assembly with the greatest *N*_50_ value is selected for further analysis. Contigs (either those assembled by virMine or those from the supplied long-read or assembled-sequence fasta file) can be filtered. This step is optional, and virMine filters include minimum and/or maximum contig length, minimum contig coverage, and the presence of sequences of interest. Next, virMine preforms gene prediction. Contigs are scored based on their gene content’s origin, i.e., cellular, viral, or unknown. virMine has successfully identified prophages and viral sequences, both homologous to known viruses and novel, from synthetic data sets and environmental samples from freshwater, the gut, and urine (3, 4).

virMine 2.0, presented here, follows the same methodology as its predecessor while incorporating updated versions of the underlying tools and databases. These updates include Python v.3.9, BBMap v.38.94 (the tool used to compute coverage statistics for the coverage filter [https://sourceforge.net/projects/bbmap]), and SPAdes v.3.15.3 (5). Furthermore, a new script to generate the virMine databases is included in this release. The script retrieves the latest bacterial Clusters of Orthologous Genes (COG) database, released in 2020 (6); it then removes all sequences of viral origin (category X) and formats the database for virMine sequence comparisons. The script also generates a viral database from the latest collection of RefSeq eukaryotic viral and phage genomes (ftp://ftp.ncbi.nlm.nih.gov/genomes/Viruses/all.faa.tar.gz) (7).

Source code and a Docker image are available at https://github.com/putonti/virmine. To use the Docker image, the user must first install the Docker application itself (https://www.docker.com). The Dockerfile builds the necessary environment with all dependencies. Once Docker is installed, the virMine repository can be cloned locally. The viral and bacterial database files can be generated using the virmine_make_dbs.py script or can be substituted with personal database files. The database files and the input files must then be transferred to the input folder within the local cloned repository. The GitHub repository provides example commands for analyses using either paired-end reads or assembled contigs. Results from the runs are saved locally within the cloned repository directory. Test data and sample output files are provided through the GitHub repository. The Docker image of virMine is also available at https://hub.docker.com/repository/docker/genevievej16/virmine; using the Docker Hub image eliminates the need to build the Docker image locally from the cloned repository.

### Data availability.

virMine and its Docker image are located online at https://github.com/putonti/virmine and https://hub.docker.com/repository/docker/genevievej16/virmine, respectively. Also included in the repository is a script to generate the updated bacterial and viral databases. Additional documentation, including setup, walkthroughs, and example commands, and test data are available in the GitHub repository.
